# Farewell to GBM-O: Genomic and transcriptomic profiling of glioblastoma with oligodendroglioma component reveals distinct molecular subgroups

**DOI:** 10.1186/s40478-015-0270-7

**Published:** 2016-01-13

**Authors:** Benjamin H. Hinrichs, Scott Newman, Christina L. Appin, William Dunn, Lee Cooper, Rini Pauly, Jeanne Kowalski, Michael R. Rossi, Daniel J. Brat

**Affiliations:** Department of Pathology and Laboratory Medicine, Emory University School of Medicine, 1648 Pierce Dr NE, Atlanta, GA 30307 USA; Biostatistics and Bioinformatics Shared Resource, Winship Cancer Institute of Emory University, 1365 Clifton Rd, Atlanta, GA 30322 USA; Department of Pathology, Northwestern University of Feinberg School of Medicine, 303 East Chicago Avenue Ward 3-140W127, Chicago, IL 60611 USA; Department of Biomedical Informatics, Emory University, 36 Eagle Row 5th Floor South, Atlanta, GA 30322 USA; Greenwood Genetics Center, JC Self Research Institute, 106 Gregor Mendel Circle, Greenwood, SC 29646 USA; Department of Biostatistics and Bioinformatics, Rollins School of Public Health, Emory University, 1518 Clifton Road, Atlanta, GA 30322 USA; Department of Radiation Oncology, Emory University School of Medicine, 1648 Pierce Dr NE, Atlanta, GA 30307 USA

**Keywords:** Glioblastoma, Glioblastoma with oligodendroglioma component, IDH mutation, 1p/19q co-deletion

## Abstract

**Introduction:**

Glioblastoma with oligodendroglioma component (GBM-O) was recognized as a histologic pattern of glioblastoma (GBM) by the World Health Organization (WHO) in 2007 and is distinguished by the presence of oligodendroglioma-like differentiation. To better understand the genetic underpinnings of this morphologic entity, we performed a genome-wide, integrated copy number, mutational and transcriptomic analysis of eight (seven primary, primary secondary) cases.

**Results:**

Three GBM-O samples had *IDH1* (p.R132H) mutations; two of these also demonstrated 1p/19q co-deletion and had a proneural transcriptional profile, a molecular signature characteristic of oligodendroglioma. The additional *IDH1* mutant tumor lacked 1p/19q co-deletion, harbored a *TP53* mutation, and overall, demonstrated features most consistent with *IDH* mutant (secondary) GBM. Finally, five tumors were *IDH* wild-type (*IDHwt*) and had chromosome seven gains, chromosome 10 losses, and homozygous 9p deletions (*CDKN2A*), alterations typical of *IDHwt* (primary) GBM. IDHwt GBM-Os also demonstrated *EGFR* and *PDGFRA* amplifications, which correlated with classical and proneural expression subtypes, respectively.

**Conclusions:**

Our findings demonstrate that GBM-O is composed of three discrete molecular subgroups with characteristic mutations, copy number alterations and gene expression patterns. Despite displaying areas that morphologically resemble oligodendroglioma, the current results indicate that morphologically defined GBM-O does not correspond to a particular genetic signature, but rather represents a collection of genetically dissimilar entities. Ancillary testing, especially for *IDH* and 1p/19q, should be used for determining these molecular subtypes.

**Electronic supplementary material:**

The online version of this article (doi:10.1186/s40478-015-0270-7) contains supplementary material, which is available to authorized users.

## Introduction

Glioblastoma (GBM) is the highest grade infiltrating astrocytoma and the World Health Organization (WHO) recognizes several morphologic patterns. Glioblastoma with Oligodendroglioma component (GBM-O), WHO grade IV, was recently described as a diffusely infiltrative, high-grade astrocytic neoplasm displaying necrosis, and also demonstrating discrete areas of oligodendroglial differentiation [1]. Some studies have suggested that oligodendroglioma-like areas in GBM may confer a favorable prognosis, yet this remains controversial [2–8]. Moreover, it is unclear whether GBM-O represents a distinct entity with defining molecular alterations and clinical behavior or a collection of genomically disparate tumors containing similar morphologic elements [2–6, 8, 9].

Prior studies suggested that GBM-O is enriched for *IDH* mutations and has fewer *PTEN* deletions than other forms of GBM [2]. Co-deletion of chromosomes 1p and 19q is the molecular signature of oligodendroglioma and associated with enhanced therapeutic response and longer survival [10], yet most studies of GBM-O have identified 1p/19q co-deletion only in a modest subset [6, 8]. Conversely, *EGFR* amplifications, which are mutually exclusive from *IDH* mutations and 1p/19q co-deletions, have been documented in GBM-Os and may define a clinically relevant subset [3]. Advanced whole genome molecular platforms can provide additional biologic insights into tumor subtypes, above that offered by morphology or a limited biomarker panel [4, 11, 12]. Therefore, in order to better characterize GBM-O, we performed copy number microarray, whole transcriptome RNA-sequencing and gene panel deep sequencing.

## Materials and methods

### Patient information and histopathology

**Fig. 1:**
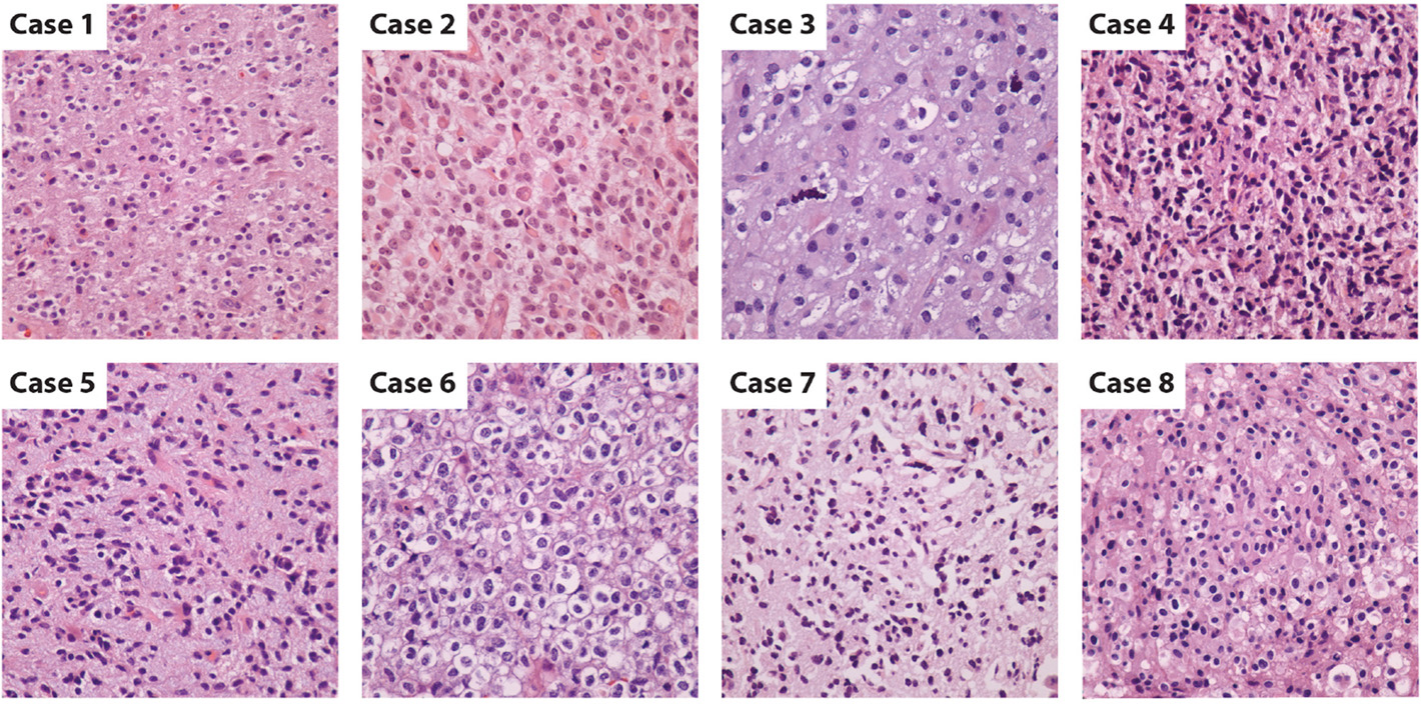
Photomicrographs of GBM-O cases. Images show representative areas of macrodissected tumor (H&E, 20×). In all cases, submitted tissue included regions of oligodendroglial differentiation

**Fig. 2:**
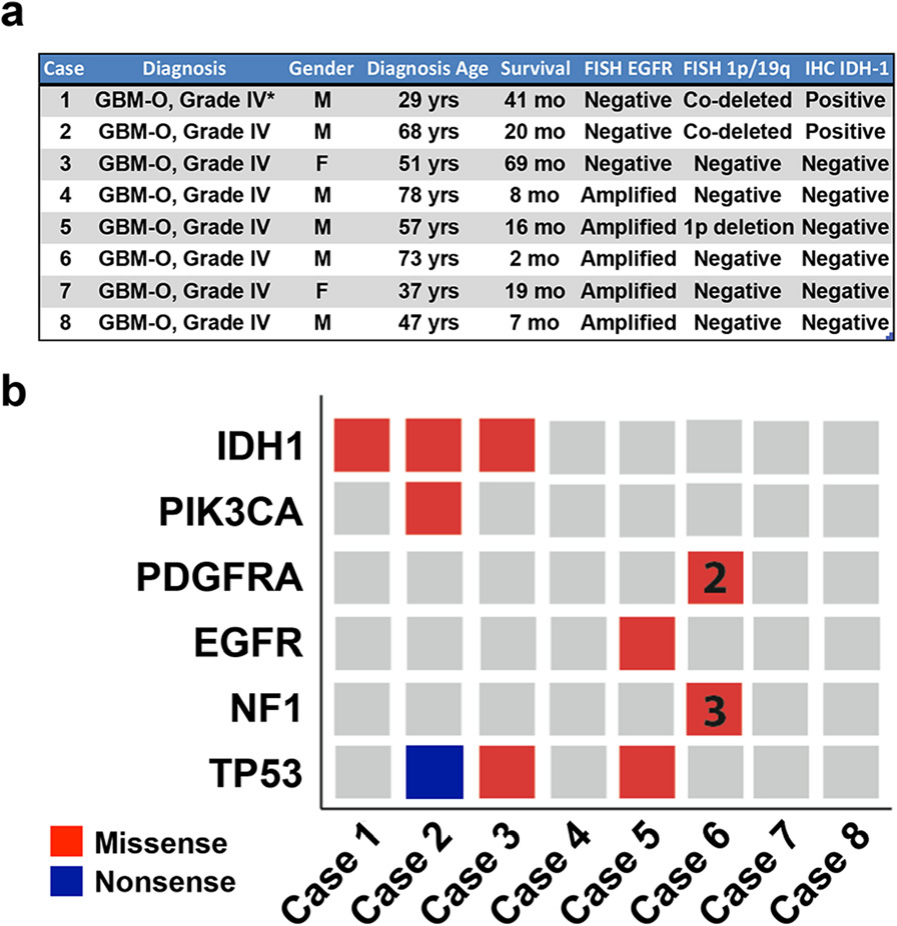
Clinicopathologic features of GBM-O cases and results of targeted Fluidigm DNA sequencing. **a** Case details including previously obtained FISH (*EGFR,* 1p/19q) and IDH1 R132H immunohistochemistry results. *prior diagnosis was fibrillary astrocytoma, grade II **b** Nonsynonymous mutations were detected in 5/8 cases (12 missense, 1 nonsense). Numerals in boxes indicate number of mutations if more than one

All tumor tissue was obtained from patients who underwent surgical resection and pathological evaluation at Emory University Hospitals from 2007 through 2011. Two neuropathologists reviewed all hematoxylin and eosin (H&E)-stained slides from each case concurrently using a multi-headed microscope. GBM-O was the original pathologic diagnosis in all cases, and all diagnoses were rendered using World Health Organization 2007 criteria [1]. Paraffin embedded tissue sections of 10-micron thickness were macro-dissected to maximize viable tumor submitted for genomic analysis. Six of eight samples contained >95 % viable tumor, while a single case each contained significant necrosis or normal tissue (25 %, case 8; 40 %, case 5, respectively). In all cases, submitted tissue included regions of oligodendroglial differentiation (Fig. 1). Summary clinical data and molecular/cytogenetic testing results are shown in Fig. 2a. FISH testing (*EGFR*, 1p/19q) and mutant IDH1 expression by immunohistochemistry, were previously performed as described [2]. Immunohistochemistry for ATRX (1:100; Sigma-Aldrich Corp., St. Louis, MO, USA) assessed nuclear expression in select cases.

### Nucleic acid isolation and QC

Serial sections were evaluated for tumor proportion and necrosis prior to nucleic acid extraction. DNA and RNA were extracted using the QIAGEN (Valencia, CA) AllPrep FFPE kit. Genomic DNA was quantitated using the Qubit dsDNA high sensitivity (HS) kit (ThermoFisher Scientific, Waltham, MA); total RNA was quantitated and quality checked using the Agilent 2100 Bioanalyzer with the RNA 6000 pico kit (Agilent Technologies, Santa Clara, CA).

### Targeted amplicon DNA sequencing

A custom amplicon-library preparation assay was developed for the Fluidigm Access Array (Fluidigm Corporation, South San Francisco, CA) using the D3 Assay Design software. Coding exons of 12 genes previously reported as recurrently mutated in GBM were targeted (*BRAF, EGFR, IDH1, IDH2, NF1, NRAS, PDGFRA, PIK3CA, PIK3R1, PTEN, RB1, TP53*) [13] (Additional file 1: Table S1). Briefly, the Bioinformatic variant/indel (collectively referred to as “variants”) pipeline comprised trimming low quality bases and adapters using Trimmomatic 0.32 [14]; merging overlapping paired end reads with ea-utils fastq-join version 1.1.2-686 (https://code.google.com/p/ea-utils/), and keeping the highest quality base call from the overlapping region; alignment to the hg19 human reference genome using the BWA-mem aligner version 0.7.5a [15]; calling potential variants with VarScan2 requiring at least 20× coverage, five non-reference reads with a minimum base quality score of 20 and a minimum variant allele frequency of 0.25 [16]. Predicted variants were annotated with ANNOVAR [17] (Additional file 2: Table S2). Since we used archival FFPE tissue without matched normal DNA, we carried out extensive filtering of predicted variants and manually verified variants using Integrative Genomics Viewer [18]. Variants not present in the Catalogue of Somatic Mutations in Cancer (COSMIC) database were required to have 1,000× coverage [19, 20]. Likely germline variants that were observed at greater than 1 % frequency in either the Thousand Genomes Project or Exome Variant Server were filtered out [21, 22]. Those found in dbSNP without previously being linked to cancer were also removed [23].

### Copy number microarray

Extraction and hybridization of genomic DNA to Illumina HumanCytoSNP-12v2.1-FFPE SNP arrays was according to manufacturer’s instructions. Data were processed and analyzed with BioDiscovery Nexus software (Hawthorne, CA) using SNPRank segmentation and a segmented log2 ratio of 0.09 to call gains, 0.3 for amplifications, -0.135 for losses and -0.45 for homozygous deletions. Combined segmented data was viewed using Copy Number Explorer software [24].

### Whole transcriptome sequencing

RNAseq library preparation and Illumina HiSeq sequencing were carried out by Beckman Coulter Genomics (Danvers, MA) using Illumina TruSeq RNA library preparation and HiSeq2500 sequencing. RNA sequencing produced approximately 50 million reads per sample. We aligned paired-end fastq files to the hg19 human reference genome using Tophat 2.0.6 under standard parameters [25]. RefSeq Transcripts were quantified and transformed into FPKM values using Cufflinks 2.02 [26].

### Gene expression-based molecular classification

We used TCGA GBM samples to derive an RNASeq gene list with which to classify our GBM-O samples. 164 TCGA samples had been classified into one of four subgroups based on expression microarray profiles and also had accompanying RNA-Seq data [13]. We initially performed non-negative matrix factorization consensus clustering with a k-value of four using gene pattern software [27] and the NMF module. We omitted samples whose NMF cluster and microarray expression subclass were discordant, leaving 115 samples. We used shrunken centroids, deployed through the pamr R-package to identify the gene sets that best classified each subtype [28]. The best-performing classification sets were a subset of the original 840-gene classifier described by Verhaak et al. [29] and comprised of 129 genes specific to classical, 160 messenchymal, 65 neural and 131 proneural genes (Additional file 3: Table S3). We used Gene Set Variation Analysis (GSVA) [30], an enhanced version of Single Sample Gene Set Enrichment Analysis (ssGSEA) [31], to classify expression microarray profiles from TCGA patient samples. GSVA classified 94 % of samples correctly (as assessed by cluster membership based on GSVA score) (not shown). We deployed GSVA through the GSVA R-package [30], to our GBM-O samples.

## Results

### Clinicopathologic features of GBM-O

From January, 2008 through December, 2011, we made the diagnosis of GBM-O 28 times. Eight of these cases were chosen based on availability of tissue and diversity of clinical diagnostic markers. Of these, seven were primary and one secondary (case 1), the latter having been diagnosed as a fibrillary astrocytoma, grade II four years prior. Ages at diagnosis ranged from 29- to 78-years (mean, 55-years) and clinical follow-up revealed patient survivals ranged from 2- to 69-months (mean, 23 months; median, 17.5 months). Tumors were classified as GBM-O if a discrete area of oligodendroglial differentiation (occupying at least a single 100× microscopic field) occurred in the setting of a high-grade astrocytic neoplasm with necrosis [2]. Oligodendroglial differentiation was recognized as glioma cells containing round, regular nuclei with only mild variation accompanied by a delicate capillary vascular network and occasionally by cytoplasmic clearing [32]. Alternatively, high-grade astrocytic differentiation was defined as glioma cells with elongated, hyperchromatic nuclei and irregular nuclear contours [32]. Photomicrographs in Fig. 1 show representative areas of tumor analyzed for each case.

Previous diagnostic testing included FISH analysis of *EGFR* and 1p and 19q as well as immunohistochemistry for IDH1 p.R132H (Fig. 2a). Cases harboring different combinations of genetic alterations were chosen to capture the molecular diversity displayed in GBM-O. Two were positive for mutant IDH1 protein and had 1p/19q co-deletions and were negative for *EGFR* amplification. Four cases were *EGFR*-amplified and did not show mutant IDH1 protein expression or 1p/19q co-deletion; an additional case was *EGFR*-amplified, IDH1 wild-type and had a 1p deletion. The remaining case did not show any molecular alteration detected by FISH or IHC staining. Of note, the presence of 1p/19q co-deletion did not correlate with higher percentages of oligodendroglioma component.

### Detection of coding variants using targeted Fluidigm DNA sequencing

We performed DNA sequencing to identify coding variants in 12 genes recurrently mutated in GBM (*BRAF, EGFR, IDH1, IDH2, NF1, NRAS, PDGFRA, PIK3CA, PIK3R1, PTEN, RB1, TP53*) [13] (Fig. 2b, Additional file 2: Table S2). In agreement with IHC results, cases 1 and 2 displayed *IDH1* (c.395G > A; p.R132H) mutations. Case 2, which was 1p/19q co-deleted, also contained a *PIK3CA* mutation, concordant with previous studies showing correlation among these alterations [11]. Additionally, case 3 also had an p.R132H mutation, despite being reported as negative by R132H IHC [33, 34], as well as a *TP53* mutation (c.481G > A, p.A161T) that has been previously reported in diffuse gliomas (COSM10739). The co-occurrence of *TP53* and *IDH* mutations is seen in the large majority of lower-grade (WHO grades II-III) diffuse astrocytomas, as well as secondary GBMs (WHO grade IV) and defines a lineage of *IDH* mutated tumors distinct from 1p/19q co-deleted oligodendrogliomas [11, 34]. Although *ATRX* alterations are frequently seen together with *IDH* and *TP53* mutations, we did not demonstrate loss of ATRX expression by IHC in this case [35, 36].

The remaining five cases were all *IDH* wild-type (*IDH*wt). *EGFR* and *PDGFRA* mutations were noted exclusively in *IDH*wt GBM-Os and all *NF1* mutations were also noted in this subset. *EGFR* and *PDGFRA* mutations were both seen in the context of *EGFR* and *PDGFRA* amplification respectively (see below), an association previously observed in primary GBM [29].

### Copy number microarray

Detection of specific somatically-acquired copy-number aberrations (CNAs) aids in CNS tumor classification, and may provide important insight into tumor biology [29]. To determine if GBM-O displayed distinct patterns of CNA, we performed copy number microarray analysis. While FISH is used diagnostically to assess CNAs at defined loci (*EGFR, PTEN* and 1p/19q) microarray testing can detect copy number changes genome wide. A full list of copy number segments is in Additional file 4: Table S4.

**Fig. 3:**
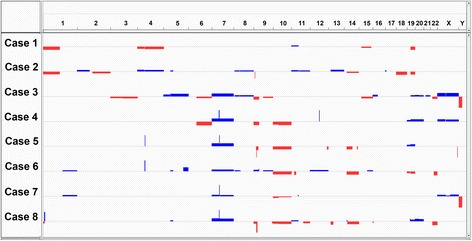
Patterns of recurrent copy-number aberrations (CNAs) identified using Illumina microarray. Focal amplifications of 7p11.2 (*EGFR*) and gains of chr7 were present in 4/8 and 6/8 cases, respectively, and were mutually exclusive of 1p/19q co-deletion and *IDH1* R132H mutation (cases 1 and 2). Loss of chr10 (*PTEN)* and homozygous 9p21.3 loss (*CDKN2A)* occurred in 4/4 and 2/4 cases with *EGFR* amplification, respectively. Focal gains involving 4q12 (*PDGFRA*) were present in 2 cases

There was general agreement between microarray and diagnostic FISH testing (Fig. 3): Cases 1 and 2 showed 1p/19q deletion by both FISH and microarray. Case 3 was negative by FISH and microarray for 1p/19q deletion and *EGFR* amplification. Cases 4, 5, 7 and 8 were *EGFR* amplified by both FISH and microarray. Case 6 was *EGFR* amplified by FISH, but the reported percentage of cells showing amplification (28/200, 14 %) was below our detection threshold of approximately 30 % for this microarray. Case 5 demonstrated 1p deletion and 19q gain by FISH. Microarray showed a gain of chromosome 19, but no loss of 1p. Reanalysis with a more sensitive segmentation algorithm, ASCAT [37, 38] (data not shown) identified a terminal 55 Mb deletion of 1p. These results are consistent and rule out a whole arm loss of chromosome 1.

Gains, losses and amplifications of other loci were evident but there was no single CNA found across all cases; rather, three general patterns were apparent: One group (cases 1 and 2) had 1p/19q co-deletion, and lacked gains of chromosome 7, amplifications of *EGFR* or losses of chromosome 10. Another group (cases 4, 5, 6, 7, and 8) demonstrated either broad gains of chromosome 7 or focal amplifications of 7p11.2 including *EGFR*, and most showed loss of chromosome 10 (including *PTEN*) as well as heterozygous deletion of 9p with or without focal homozygous deletion of *CDKN2A*. Several of these cases also had other CNAs previously described in GBM but not oligodendroglioma including *PDGFRA* amplification (cases 5 and 6) and *MDM2* amplification (case 4) [13]. Lastly, case 3 displayed a third pattern of copy number alteration. While showing alterations overlapping those seen in the latter group (gain of chromosome 7 and heterozygous deletion of 9p), case 3 also demonstrated alterations not present in other cases including loss of chromosome 3 and 9q, as well as gains of 16p and chromosomes 5 and 21.

### Gene expression-based molecular classification

**Fig. 4:**
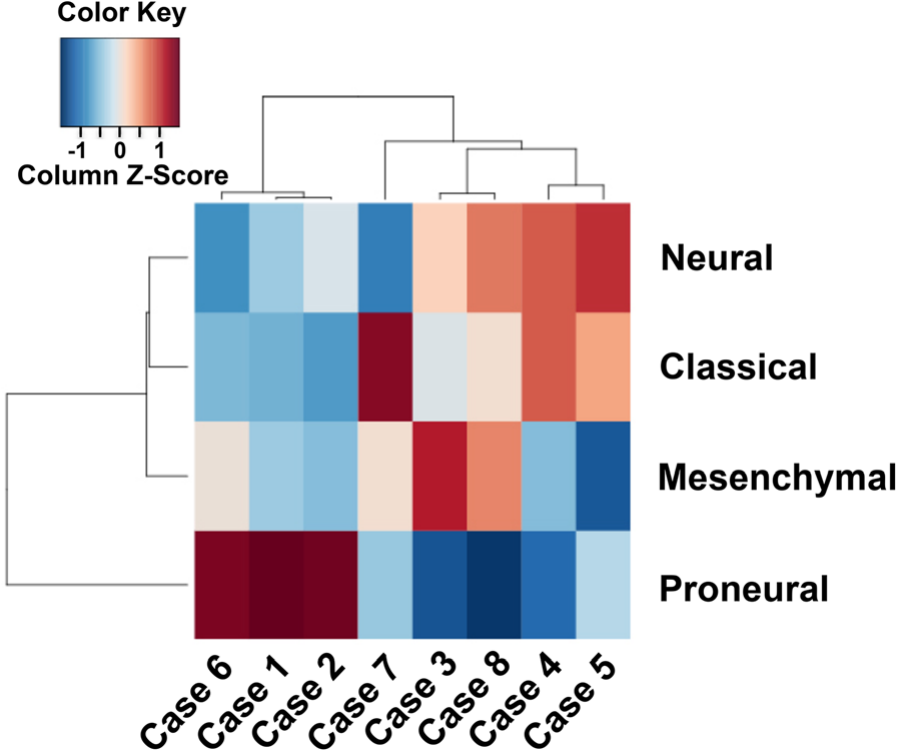
Gene expression profile heat map. A positive enrichment score (column Z-score) indicates the tumor sample expression profile and the genes in that particular gene set are positively correlated, whereas a negative enrichment score indicates the opposite. Five tumors demonstrated expression signatures enriched for one subtype, while the remaining three had transcriptional profiles enriched for two. Three tumors showed exclusive classification as the proneural subtype (cases 1, 2, and 6), and one tumor each was classified exclusively as classical and mesenchymal (cases 7 and 3, respectively). Cases 4 and 5 showed both classical and neural signatures, while case 8 showed both mesenchymal and neural subtype expression patterns

Previous gene expression studies identified four GBM expression subtypes - proneural, classical, neural and mesenchymal - named after the expression signature’s resemblance to transcriptional profiles of neural cell types [29]. We next determined if our GBM-O samples were all classified as the same expression subgroup or, alternatively, represented a combination of two or more subgroups. We performed RNASeq gene expression profiling and classified these data using Gene Set Variation Analysis (GSVA). GSVA expression enrichment scores for GBM-O cases are displayed in Fig. 4. A column Z-score of >0.5 was used as a cutoff to determine whether a tumor sample was enriched for a particular subtype expression signature. Additional file 5: Figure S1 displays nominal scores indicating correlation between sample transcriptional profile and expression subtype compared across samples. Expression values for select genes are provided in Additional file 6: Table S5.

Overall, all four GBM expression subtypes were represented among the eight GBM-Os (Fig. 4). Five demonstrated expression signatures strongly enriched for one subtype, while the remaining three had transcriptional profiles enriched for two subtypes (Fig. 4 and Additional file 5: Figure S1). Three tumors showed exclusive classification as the proneural subtype (cases 1, 2, and 6), and one tumor each was classified exclusively as classical and mesenchymal (cases 7 and 3, respectively). Cases 4 and 5 showed enrichment for both classical and neural genes, while case 8 showed both mesenchymal and neural signatures.

The three tumor samples classified as proneural were tightly clustered and included two of three *IDH1* mutant tumor samples, consistent with other studies demonstrating this strong association [3, 29]. Also in concordance with previous studies, all cases demonstrating proneural gene expression signatures showed increased expression levels of the proneural gene *DCX* [29]. An additional GBM-O with proneural expression profile exhibited high-level *PDGFRA* amplification and gene expression, also typical of this transcriptional subtype [29].

Cases with classical signatures demonstrated substantially elevated *EGFR* expression, a discriminating feature of this subtype [29]. Consistent with previous reports, tumor samples in the classical subgroup also demonstrated increased expression of *LFNG,* a gene encoding for an O-fucosylpeptide 3-beta-N-acetylglucosaminyltransferase known to affect Notch signaling [29].

Two cases showed enrichment for the mesenchymal gene set and both demonstrated increased expression of *CHI3L1*, a marker consistently elevated in this subtype [39]. *RELB* and *TNFRSF1A*, genes involved in the NF*-*kB and tumor necrosis factor super family pathways, were also elevated, consistent with prior reports [29]*.* One of the GBM-Os classified as mesenchymal (case 8) demonstrated a significant percentage of necrosis (25 %), a feature observed in mesenchymal GBMs in the TCGA cohort [29].

Three GBM-O samples demonstrated enrichment for neural gene sets and two of the three showed substantially elevated expression of the neuron marker *SYT1* in concordance with previous studies [29]. Although two normal brain tissue samples analyzed by Verhaak et al. [29] were categorized as the neural subtype, only one of the three current tumor samples contained a significant percentage of benign tissue (case 5).

### Integration of molecular platforms

**Fig. 5:**
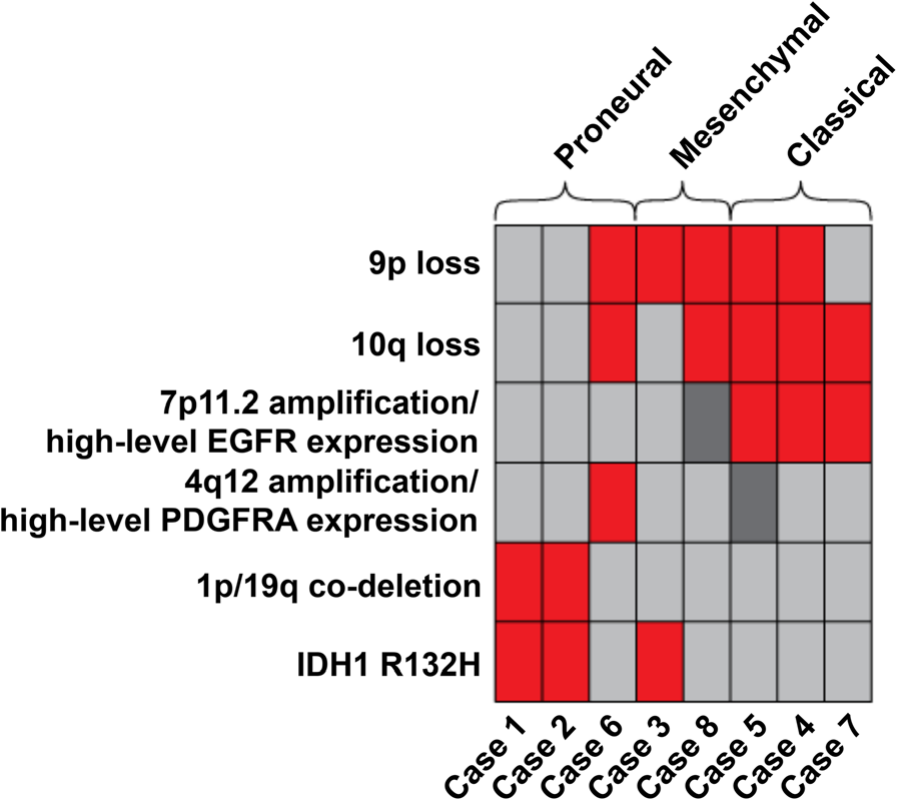
Overview of molecular aberrations in GBM-O cases. Each column represents a single case and cases are grouped by expression signature subtype. In regards to *EGFR* and *PDGFRA* alterations, *red boxes* indicate samples demonstrated both DNA amplification and high-level gene expression, whereas samples with *dark grey boxes* showed only amplification. *Light grey boxes* indicate negative for mutation or balanced chromosomal status

Previous studies of GBM have shown associations between transcriptional signatures, somatic mutations and DNA copy number alterations [3, 29]. To investigate whether these associations are also present in GBM-O, we correlated patterns of somatic mutation and DNA copy number alterations with expression subtype (Fig. 5). Overall, the selected GBM-Os could be divided into three groups. The first (cases 1 and 2) consisted of GBM-Os with *IDH1* mutations and 1p/19q co-deletions and lacked DNA copy number alterations typical of GBM such as *EGFR* amplification, gains of chromosome 7 and losses of 10 [13]. *IDH1* mutations and 1p/19q co-deletion have been shown to be strongly correlated in GBM-O [2, 40]. Case 2 also contained a *PIK3CA* mutation, a finding previously documented with *IDH* mutations and 1p/19q co-deletion [11]. Both *IDH1* mutant, 1p/19q co-deleted tumors showed a proneural gene expression signature, and, therefore, displayed genetic alterations resembling those seen in oligodendrogliomas [34]*.*

An additional tumor (case 3) was *IDH1* mutated, but lacked 1p/19q co-deletion and harbored a *TP53* mutation. This genetic pattern is typical of *IDH* mutant diffuse atrocytomas of WHO grades II, III and IV [41]. While this case showed additional characteristics of astrocytic lineage, including gain of chromosome 7 and heterozygous deletion of 9p, it lacked typical alterations of primary GBM such as *EGFR* amplification, chromosome 10 loss and homozygous deletion of *CDKN2A*. Taken together, these findings are most consistent with an *IDH* mutant GBM, often referred to clinically as “secondary” GBM.

The remaining 5 GBM-Os were *IDH *wild-type (*IDH*wt). Four of the *IDH*wt tumors were amplified for *EGFR* (cases 4, 5, 7 and 8), had broad gains of chromosome 7, loss of chromosome 10, *CDKN2A* deletion (two cases) and showed enrichment for the classical expression gene sets (three cases). Among cases with classical transcription signatures, high-level *EGFR* amplification corresponded to significantly elevated *EGFR* expression. The GBM-O that harbored an *EGFR* mutation also showed *EGFR* amplification and had a classical expression signature.

One of the *IDH*wt tumors (case 6) demonstrated a proneural expression signature and exhibited the characteristic constellation of 4q12 focal amplification involving *PDGFRA*, high levels of *PDGFRA* gene expression, as well as *PDGFRA* mutation. This case also demonstrated chromosome 7 gain and losses of 10 and 9p, indicating additional copy number similarity with primary GBM.

Among *IDH*wt tumors, transcriptional profiles were predominantly classified as classical and neural; however, this subgroup also contained representatives from the proneural and mesenchymal subtypes. *IDH*wt GBM-Os in the current study, therefore, displayed gene expression diversity similar to GBMs in the TCGA cohort [29]. Coupled with the classic DNA copy-number alterations discussed above, the *IDH*wt subgroup overall demonstrated molecular alterations most consistent with primary GBM.

## Conclusions

Glioblastoma with Oligodendroglioma component (GBM-O) was introduced by the WHO classification in 2007 as one of the morphologic patterns of GBM [1]. Clinical outcomes data and biomarker profiles have, however, yielded inconsistent results and the clinical significance and biology of GBM-O is not well understood. While morphology has informed CNS tumor classification for decades, molecular testing can also define distinct genetic entities with specific clinical outcomes. Therefore, we employed copy number microarray, whole transcriptome RNA-Sequencing and gene panel deep sequencing to better characterize the GBM-O entity. Our results showed that despite having overlapping morphologic features, our GBM-O cohort was composed of three discrete molecular subgroups, each with characteristic coding variants, CNAs and gene expression patterns.

Previous investigations have also attempted to clarify the significance of GBM-O through molecular testing. The presence of 1p/19q co-deletions has received particular attention given its association with oligodendrogliomas and improved clinical outcomes [41]; however, reported frequencies having ranged from 4 to 30 % [2–6, 8]. Compared with conventional GBM, GBM-Os show consistently increased frequencies of *IDH1* mutations, though enrichment of GBM-O cohorts with secondary GBMs may explain this finding [2, 3, 8]. Despite the evidence for an increased association between GBM-O and *IDH* mutations and 1p/19q co-deletions, there is variation in clinical outcome studies with some showing a favorable prognosis when compared to conventional GBMs [2, 6–8, 42], while others do not [3–5]. Lastly, investigations show wide-ranging frequencies of *EGFR* amplification (39–71 %) as well as LOH of 10q (0–58 %) highlighting the molecular heterogeneity seen in GBM-O [2, 3, 6, 42]. Our current data indicates that the variations in clinical outcomes and biomarker status reported in these investigations may be due to the inclusion of multiple molecular genetic entities with overlapping morphologic features into the analyses. While additional molecular variation could potentially exist in the setting of morphologic diversity, previous data has indicated genomic stability across morphologic phenotypes [5].

A crucial subdivision of diffuse gliomas is based on *IDH* mutational status, since *IDH* mutant tumors progress more slowly and are associated with prolonged survival, grade for grade [11, 34]. The large majority of grades II and III diffuse gliomas, including oligodendrogliomas and astrocytomas, are *IDH* mutant [11]. Only a small minority of GBMs are *IDH* mutant and many of these have progressed from grade II and III astrocytomas (secondary GBMs) [13]. In our analysis, three of eight GBM-Os were *IDH* mutant, whereas five were *IDH*wt. Two of the *IDH* mutant tumors in our cohort were 1p/19q co-deleted recapitulating the strong positive correlation between these alterations seen in other studies of GBM-O [2, 9, 40]. These tumors also had a proneural transcriptional profile, and therefore, overall demonstrated a molecular signature that is classic for oligodendroglioma. Both patients with this molecular signature had survivals (20 and 41 months) longer than the median for this cohort (17.5 months). Integrating the genetic findings of these high grade gliomas with *IDH* mutations and 1p/19q co-deletions, as suggested by a recent international consensus of neuropathologists, would lead to their categorization as anaplastic oligodendroglioma, WHO grade III, with *IDH* mutation and 1p/19q co-deletion, rather than GBM-O [43].

An additional tumor (case 3) was *IDH1* mutated, but it lacked 1p/19q co-deletion and harbored a *TP53* mutation. The constellation of *IDH1* and *TP53* mutation without 1p/19q co-deletion is seen in diffuse astrocytomas of WHO grade II, III and IV [11, 41]. Interestingly, the occurrence of a *TP53* mutation in the context of an intact 1p arm as in this case, is consistent with the mutual exclusivity of 1p deletion and p53 overexpression observed in other GBM-O cohorts [2, 9]. Typical alterations seen in *IDH*wt (primary) GBM such as *EGFR* amplification, chromosome 10 loss and homozygous deletion of *CDKN2A* were not present, although the observed gain of chromosome 7 and heterozygous deletion of 9p provide further evidence of the tumor’s astrocytic lineage. Overall, the identified *IDH1* and *TP53* mutations, the lack of molecular alterations seen in primary GBM and the longest survival of the study indicate this case is an *IDH* mutant form of GBM, often referred to as “secondary GBM”.

*IDH* wild-type GBM-Os in our cohort, on the other hand, were characterized by chromosome 7 gains, chromosome 10 losses, and 9p (*CDKN2A*) deletions; signatures of an astrocytoma lineage and most typical of *IDH*wt GBM [29, 41]. This subgroup contained additional alterations characteristic of *IDH*wt GBM such as *EGFR* amplification and mutation, as well as *NF1* mutations. *EGFR* amplified tumors were mostly associated with the classical transcriptional class, as previously described [29]. *IDH*wt GBM-Os also contained representatives from the proneural, mesenchymal, and neural subtypes and, therefore, display diversity homologous to GBMs in the TCGA cohort [29]. In addition, we also identified an *IDH*wt GBM-O with high level *PDGFRA* amplification and a gene expression profile aligned with the proneural class, an association also previously established in primary GBM [29]. Lastly, consistent with behavior of tumors molecularly resembling primary GBM, all *IDH*wt cases had survivals shorter than the mean. Average patient survival of this group, however, did not differ significantly from *IDH* mutant tumors in this small cohort.

Recent investigations of the diffuse gliomas have indicated that *IDH* mutations and 1p/19q co-deletion provide a framework for defining molecular classes that distinguish lineages and clinical behavior [11, 41]. Diffuse gliomas with *IDH* mutations and 1p/19q co-deletion correspond to oligodendrogliomas, while *IDH* mutant tumors lacking the co-deletion typically have *TP53* mutations, *ATRX* alterations and are considered astrocytic in differentiation [11, 34, 41]. Diffuse gliomas that are *IDH*wt are typically high grade and have clinical behavior and genetic alterations similar of primary GBM [11, 13, 41]. Lastly, grade II and III diffuse gliomas that contain morphologic features of both oligodendroglioma and astrocytoma, i.e. oligoastrocytoma, have now been shown to have either the molecular signature of oligodendroglioma or astrocytoma [44]. Thus, some have questioned the existence of oligoastrocytoma at the molecular level [44, 45]. Combining the above molecular-based approach with morphologic appearance has led to the use of an “integrated” diagnostic framework. Applied specifically to tumors diagnosed as GBM-O, recent studies have shown this diagnostic algorithm separates GBM-O into prognostically relevant groups, namely anaplastic oligodendroglioma and GBM, with the latter further classified based on *IDH* mutation status [36, 46]. The overall patterns of genetic alterations that we have noted in GBM-O suggest a similar conclusion and indicate that these tumors can be divided into three discrete molecular classes: 1) those that are *IDH* mutant and 1p/19q co-deleted have the molecular signature of a high grade oligodendroglioma; 2) those that are *IDH* mutant and not co-deleted have features of secondary GBM; and 3) those that are *IDH*wt have the molecular signature of primary GBMs.

Therefore, despite displaying areas that morphologically resemble oligodendroglioma, the current findings indicate that these foci do not specifically signify the presence of 1p/19q co-deletion, and overall lack specificity for a particular genetic signature. Taken together, the findings suggest molecular testing should be relied on for establishing diagnoses of diffuse gliomas.

## Availability of data and materials

The datasets supporting the conclusions of this article are included within the article and its additional files.

## Additional files

Additional file 1: Table S1. Targeted amplicon DNA sequencing coding exons. (XLSX 91 kb) Additional file 2: Table S2. Targeted amplicon DNA sequencing. (XLSX 49 kb) Additional file 3: Table S3. Gene lists for expression subtypes. (PDF 52 kb) Additional file 4: Table S4. Copy number segments. (PDF 27 kb) Additional file 5: Figure S1. Expression histogram. (TIFF 3115 kb) Additional file 6: Table S5. Expression values for select genes. (XLSX 148 kb)
