# Supplementary material for: Farewell to GBM-O: Genomic and transcriptomic profiling of glioblastoma with oligodendroglioma component reveals distinct molecular subgroups
Source: Acta Neuropathol Commun. 2016 Jan 13;4:4. doi: 10.1186/s40478-015-0270-7 (PMC4711079; doi:10.1186/s40478-015-0270-7)
Supplement: Additional file 3: Table S3. — Gene lists for expression subtypes. (PDF 52 kb) [file 40478_2015_270_MOESM3_ESM.pdf]

Gene	Result_Classification
UCP2	Classical
EPB41L3	Classical
PPA1	Classical
ENPP2	Classical
MMD	Classical
SLC31A2	Classical
ENPP4	Classical
EDIL3	Classical
TLR4	Classical
RBM42	Classical
KHSRP	Classical
NR2E1	Classical
GTF2F1	Classical
DOT1L	Classical
NCLN	Classical
LAMA5	Classical
CDK6	Classical
C19orf22	Classical
CHERP	Classical
GANAB	Classical
ARAP2	Classical
UPF1	Classical
DLC1	Classical
HSPBP1	Classical
DMWD	Classical
JUND	Classical
PLEKHA4	Classical
LRRC16A	Classical
KLHL25	Classical
CC2D1A	Classical
ZNF146	Classical
VPS16	Classical
FGFR3	Classical
TYK2	Classical
LRP5	Classical
SLC6A9	Classical
SLC6A11	Classical
MYST2	Classical
MLEC	Classical
SEZ6L	Classical
SLC12A4	Classical
SCG3	Classical
QTRT1	Classical
MYO9B	Classical
PRPF31	Classical
POMT2	Classical
MEOX2	Classical

PEPD		Classical
UNC45A		Classical
TCF3		Classical
CDH4		Classical
MCC		Classical
SIPA1L1		Classical
BLM		Classical
ACSS3		Classical
LMO2		Classical
EYA2		Classical
PTBP1		Classical
KLHL4		Classical
NPEPL1		Classical
CREB5		Classical
CAMK2B		Classical
SOX2		Classical
ARNTL		Classical
AP3D1		Classical
RGS12		Classical
ADAM19		Classical
RBCK1		Classical
POFUT1		Classical
VAV3		Classical
C19orf6		Classical
CD97		Classical
GRIK5		Classical
NOS2		Classical
MEGF8		Classical
ARAP3		Classical
CD151		Classical
IRS2		Classical
LHFP		Classical
RGS6		Classical
SEMA6A		Classical
SOCS2		Classical
ACSBG1		Classical
BTBD2		Classical
TSPAN3		Classical
GLG1		Classical
SLC4A4		Classical
SCAMP4		Classical
	11-Sep	Classical
TRIO		Classical
EXTL3		Classical
ZYX		Classical
SEMA6D		Classical
MYO10		Classical
SPPL2B		Classical

SPRY2	Classical
JAG1	Classical
ITGB8	Classical
LAMB2	Classical
AKT2	Classical
PIPOX	Classical
ACTN4	Classical
NPAS3	Classical
GAS1	Classical
CKB	Classical
KLHDC8A	Classical
CALM1	Classical
DAG1	Classical
CDH2	Classical
GNAS	Classical
TRIB2	Classical
GNG7	Classical
KCNF1	Classical
PDGFA	Classical
WSCD1	Classical
ACSL3	Classical
PLCG1	Classical
GPR56	Classical
CLIP2	Classical
ITGA7	Classical
CSPG5	Classical
ELOVL2	Classical
ANXA5	Classical
NLGN3	Classical
TTYH1	Classical
MLC1	Classical
DPP6	Classical
PTPRA	Classical
LFNG	Classical
EGFR	Classical
DENND2A	Classical
SOX9	Classical
NES	Classical
SRGAP3	Mesenchymal
CDKN1B	Mesenchymal
MAN2A1	Mesenchymal
EPHB4	Mesenchymal
TRPM2	Mesenchymal
MYO1E	Mesenchymal
FHL2	Mesenchymal
SQRDL	Mesenchymal
BLVRB	Mesenchymal
TMEM43	Mesenchymal

SWAP70	Mesenchymal
PLA2G15	Mesenchymal
FPR3	Mesenchymal
LRRFIP1	Mesenchymal
YAP1	Mesenchymal
CASP4	Mesenchymal
RBMS1	Mesenchymal
EHD2	Mesenchymal
CDCP1	Mesenchymal
PTPN6	Mesenchymal
RAC2	Mesenchymal
WWTR1	Mesenchymal
UAP1	Mesenchymal
COL8A2	Mesenchymal
TNFAIP3	Mesenchymal
THBD	Mesenchymal
LAMC1	Mesenchymal
ANXA4	Mesenchymal
SEC61A1	Mesenchymal
PLIN3	Mesenchymal
SLAMF8	Mesenchymal
RRAS	Mesenchymal
SH2B3	Mesenchymal
ADAM12	Mesenchymal
PGCP	Mesenchymal
SEC24D	Mesenchymal
PABPC1	Mesenchymal
PXN	Mesenchymal
MAN1A1	Mesenchymal
ACSL4	Mesenchymal
TRIP6	Mesenchymal
IGFBP6	Mesenchymal
FMNL1	Mesenchymal
SP100	Mesenchymal
LCP2	Mesenchymal
S100A4	Mesenchymal
EMP3	Mesenchymal
LRP10	Mesenchymal
CNN2	Mesenchymal
FOLR2	Mesenchymal
ITGAM	Mesenchymal
WIPF1	Mesenchymal
CKAP4	Mesenchymal
LTBP1	Mesenchymal
MYO1F	Mesenchymal
CYTH4	Mesenchymal
STAT6	Mesenchymal
POLD4	Mesenchymal

LGALS3	Mesenchymal
PLOD3	Mesenchymal
FNDC3B	Mesenchymal
PLAU	Mesenchymal
TCIRG1	Mesenchymal
CYBRD1	Mesenchymal
P4HA2	Mesenchymal
FCGR2B	Mesenchymal
FXYD5	Mesenchymal
DSE	Mesenchymal
IL1R1	Mesenchymal
TLR2	Mesenchymal
LAMB1	Mesenchymal
IL4R	Mesenchymal
ALOX5	Mesenchymal
PTPRC	Mesenchymal
THBS1	Mesenchymal
COL1A2	Mesenchymal
LTBP2	Mesenchymal
RHOG	Mesenchymal
MS4A4A	Mesenchymal
CEBPB	Mesenchymal
C1orf38	Mesenchymal
CHPF2	Mesenchymal
EFEMP2	Mesenchymal
SERPINH1	Mesenchymal
ANXA1	Mesenchymal
TGOLN2	Mesenchymal
LOX	Mesenchymal
TYMP	Mesenchymal
COL1A1	Mesenchymal
FLNA	Mesenchymal
COL5A1	Mesenchymal
C5AR1	Mesenchymal
FZD7	Mesenchymal
DAB2	Mesenchymal
ACSL1	Mesenchymal
GLT25D1	Mesenchymal
DCBLD2	Mesenchymal
LMAN1	Mesenchymal
TPM4	Mesenchymal
PYGL	Mesenchymal
TGFBI	Mesenchymal
ENG	Mesenchymal
TMBIM1	Mesenchymal
LGALS1	Mesenchymal
HSP90B1	Mesenchymal
PLS3	Mesenchymal

FURIN	Mesenchymal
CTSA	Mesenchymal
SRPX2	Mesenchymal
CLIC1	Mesenchymal
MYH9	Mesenchymal
SERPINE1	Mesenchymal
SLC11A1	Mesenchymal
MYOF	Mesenchymal
LAIR1	Mesenchymal
PTRF	Mesenchymal
SCPEP1	Mesenchymal
CAST	Mesenchymal
LCP1	Mesenchymal
SLC16A3	Mesenchymal
MFSD1	Mesenchymal
MAFB	Mesenchymal
PLAUR	Mesenchymal
TNFRSF1B	Mesenchymal
MVP	Mesenchymal
ITGA5	Mesenchymal
TGFBR2	Mesenchymal
LHFPL2	Mesenchymal
MGAT1	Mesenchymal
MSR1	Mesenchymal
IQGAP1	Mesenchymal
MRC2	Mesenchymal
HEXB	Mesenchymal
HEXA	Mesenchymal
TPM3	Mesenchymal
MAN2B1	Mesenchymal
CD4	Mesenchymal
IFI30	Mesenchymal
TNFRSF1A	Mesenchymal
P4HB	Mesenchymal
FCGR2A	Mesenchymal
CTSC	Mesenchymal
SAT1	Mesenchymal
SHC1	Mesenchymal
NPC2	Mesenchymal
NRP1	Mesenchymal
CD14	Mesenchymal
CHI3L1	Mesenchymal
ARPC1B	Mesenchymal
STAB1	Mesenchymal
SERPINA1	Mesenchymal
S100A11	Mesenchymal
CTSZ	Mesenchymal
ANXA2	Mesenchymal

ITGB2	Mesenchymal
GRN	Mesenchymal
LAPTM5	Mesenchymal
TIMP1	Mesenchymal
CTSB	Mesenchymal
NCL	Neural
KPNB1	Neural
CHD4	Neural
NCOR2	Neural
SSRP1	Neural
HNRNPUL2	Neural
LARP1	Neural
AFAP1	Neural
TRRAP	Neural
HNRNPAB	Neural
DNMT1	Neural
ABL1	Neural
SAFB	Neural
NUP188	Neural
GATAD2A	Neural
RBM15B	Neural
DDX42	Neural
PLXNA1	Neural
HCFC1	Neural
CDV3	Neural
ELAVL1	Neural
PRKDC	Neural
TOP1	Neural
TPR	Neural
XPO6	Neural
COL4A2	Neural
GCN1L1	Neural
BRD4	Neural
PPM1G	Neural
RBM10	Neural
EEF2	Neural
KDM2A	Neural
PEX19	Neural
SERPINI1	Neural
C1orf54	Neural
MGST2	Neural
CRYM	Neural
PHF11	Neural
RAB32	Neural
SNTA1	Neural
NSL1	Neural
ANKRD46	Neural
TSNAX	Neural

LYRM1	Neural
PMP22	Neural
GSTK1	Neural
TCEAL1	Neural
MRPL49	Neural
CAMK2G	Neural
TTC1	Neural
MORF4L2	Neural
CHN1	Neural
NDUFS3	Neural
HPCA	Neural
MAT2B	Neural
ADD3	Neural
VAMP5	Neural
IMPA1	Neural
FAM49B	Neural
SYNGR2	Neural
HPCAL4	Neural
EVI2A	Neural
NDRG2	Neural
TMEM144	Neural
ANXA7	Neural
NDP	Neural
CRBN	Neural
ACYP2	Neural
BEX1	Neural
SYPL1	Neural
CRYL1	Neural
MDH1	Neural
MYBPC1	Neural
CDC42	Neural
COX5B	Neural
ATP5F1	Neural
GUK1	Neural
ATP5L	Neural
SEPP1	Neural
YPEL5	Neural
MGST3	Neural
SCHIP1	Neural
S100A13	Neural
GABARAPL2	Neural
SEPW1	Neural
GJA1	Neural
CALM2	Neural
TRIM22	Proneural
OSBPL3	Proneural
ILK	Proneural
SLC2A10	Proneural

PDPN	Proneural
PLA2G5	Proneural
TGFB3	Proneural
ZNF217	Proneural
CCDC109B	Proneural
C19orf66	Proneural
FAM38A	Proneural
TMEM147	Proneural
ARSJ	Proneural
ARHGAP29	Proneural
CYTH1	Proneural
VEZF1	Proneural
RALGPS1	Proneural
SEC61A2	Proneural
ZNF286A	Proneural
C1orf61	Proneural
SH3GL2	Proneural
SRRM2	Proneural
BCOR	Proneural
REEP1	Proneural
HMGB3	Proneural
KIAA1598	Proneural
FBXO21	Proneural
PHLPP1	Proneural
DUSP26	Proneural
TCEAL2	Proneural
TMEM35	Proneural
ANKS1B	Proneural
HNRNPM	Proneural
C1orf106	Proneural
TOX3	Proneural
ICK	Proneural
DPF1	Proneural
KDM4B	Proneural
TMCC1	Proneural
PELI1	Proneural
FUT9	Proneural
FAM110B	Proneural
RBPJ	Proneural
ZNF711	Proneural
CASK	Proneural
LRP6	Proneural
MMP15	Proneural
P2RX7	Proneural
LMNB2	Proneural
ASCL1	Proneural
HNRNPH3	Proneural
CAMSAP1L1	Proneural

GSK3B	Proneural
GNAI1	Proneural
NRXN1	Proneural
GADD45G	Proneural
ARHGEF9	Proneural
TMSB15A	Proneural
RAB33A	Proneural
NCALD	Proneural
TMEFF1	Proneural
ILF3	Proneural
MAST1	Proneural
MBP	Proneural
CBX1	Proneural
NKX2-2	Proneural
GSTA4	Proneural
CELF3	Proneural
CRB1	Proneural
SLC1A1	Proneural
MATR3	Proneural
HNRNPA3	Proneural
GPM6A	Proneural
MAGEH1	Proneural
RAD21	Proneural
ABAT	Proneural
DNM3	Proneural
BCL7A	Proneural
SATB1	Proneural
MYT1	Proneural
CDK5R1	Proneural
SMARCA4	Proneural
SPTBN2	Proneural
GRIA2	Proneural
CA10	Proneural
NKAIN1	Proneural
ARHGAP33	Proneural
CNTN1	Proneural
BCAN	Proneural
DCAF7	Proneural
KDM1A	Proneural
UGT8	Proneural
LPHN3	Proneural
BASP1	Proneural
FERMT1	Proneural
DLL3	Proneural
ACTR1A	Proneural
ATP1A3	Proneural
STMN4	Proneural
TOP2B	Proneural

HDAC2	Proneural
NRXN2	Proneural
WASF1	Proneural
DPYSL4	Proneural
SCN3A	Proneural
ZEB2	Proneural
BCAS1	Proneural
MAPT	Proneural
FAM125B	Proneural
CHD7	Proneural
ERBB3	Proneural
EPHB1	Proneural
RUFY3	Proneural
C6orf134	Proneural
HN1	Proneural
C1QL1	Proneural
TTC3	Proneural
GPR17	Proneural
PODXL2	Proneural
CSNK1E	Proneural
SOX11	Proneural
MLLT11	Proneural
GNG4	Proneural
FXYD6	Proneural
MTSS1	Proneural
OLIG2	Proneural
KIF21B	Proneural
DCX	Proneural
RAP2A	Proneural
MARCKS	Proneural
DBN1	Proneural
CLASP2	Proneural
PFN2	Proneural
STMN1	Proneural
MAP2	Proneural
SOX10	Proneural
AMOTL2	Proneural
ALCAM	Proneural
LPPR1	Proneural
SOX4	Proneural
NCAM1	Proneural
CRMP1	Proneural
MARCKSL1	Proneural
