# Supplementary material for: Farewell to GBM-O: Genomic and transcriptomic profiling of glioblastoma with oligodendroglioma component reveals distinct molecular subgroups
Source: Acta Neuropathol Commun. 2016 Jan 13;4:4. doi: 10.1186/s40478-015-0270-7 (PMC4711079; doi:10.1186/s40478-015-0270-7)
Supplement: Additional file 4: Table S4. — Copy number segments. (PDF 27 kb) [file 40478_2015_270_MOESM4_ESM.pdf]

GBM-1	chr1	0	10	1	-0.3236544
GBM-1	chr1	10	121184898	7913	-0.3236544
GBM-1	chr11	0	51530241	4164	0.09890347
GBM-1	chr15	20161372	90804877	4875	-0.1425811
GBM-1	chr19	27801814	59128983	1775	-0.3272553
GBM-1	chr4	0	49053522	3188	-0.2197808
GBM-1	chr4	52728324	191154276	7586	-0.2523715
GBM-2	chr1	0	121184898	7912	-0.2673412
GBM-2	chr11	0	51530241	4165	0.12401462
GBM-2	chr11	54835623	135006516	5868	0.09933405
GBM-2	chr13	19263735	115169878	7001	0.15499125
GBM-2	chr14	20213937	107349540	6294	-0.1394929
GBM-2	chr17	0	8239050	620	0.10123808
GBM-2	chr18	0	15375878	1359	-0.3214143
GBM-2	chr18	18540853	78077248	4178	-0.3141171
GBM-2	chr19	27801814	59128983	1775	-0.2888645
GBM-2	chr2	0	90247720	7134	0.14732476
GBM-2	chr2	113311633	243199373	8299	-0.1811576
GBM-2	chr4	0	49053522	3188	0.15335949
GBM-2	chr4	52728324	191154276	7588	0.13490162
GBM-2	chr5	49646456	68688185	1401	0.09523337
GBM-2	chr8	8101641	43646413	2933	0.09020276
GBM-2	chr8	47060977	146364022	7125	0.09559757
GBM-2	chr9	9118816	9817078	82	-0.6507828
GBM-3	chr15	20161372	102531392	6124	-0.1813038
GBM-3	chr16	0	35069526	2557	0.16544829
GBM-3	chr19	27801814	59128983	1775	0.12113418
GBM-3	chr20	0	26225145	2417	0.14323677
GBM-3	chr20	29843182	63025520	2602	0.12295687
GBM-3	chr21	14687571	48129895	2864	0.11082914
GBM-3	chr22	16079545	51304566	2737	-0.2210162
GBM-3	chr3	0	91000000	6039	-0.16232
GBM-3	chr3	93632198	198022430	6423	-0.1781445
GBM-3	chr5	0	46228333	3793	0.1898322
GBM-3	chr5	49646456	68688185	1401	0.24948435
GBM-3	chr5	70762935	180915260	7453	0.24472459
GBM-3	chr6	61000000	171115067	7458	-0.1537026
GBM-3	chr7	0	57789531	4679	0.28129113
GBM-3	chr7	61760895	159138663	5552	0.28058679
GBM-3	chr8	8101641	43646413	2935	0.11940029
GBM-3	chr8	47060977	146364022	7123	0.11208268
GBM-3	chr9	0	38771460	3182	-0.2910309
GBM-3	chr9	71034203	141213431	5249	-0.1369549

GBM-3	chrX	0	59336852	2945	0.31957504
GBM-3	chrX	65125757	155270560	3657	0.31182489
GBM-3	chrY	2655180	10077460	185	-2.2819514
GBM-3	chrY	13656660	19567718	388	-2.157545
GBM-3	chrY	20828795	24522333	215	-2.2514799
GBM-4	chr10	0	38685231	3187	-0.2880657
GBM-4	chr10	42810783	135534747	6465	-0.3415894
GBM-4	chr12	69078092	69329578	16	1.69959462
GBM-4	chr19	0	24556461	1585	0.13570732
GBM-4	chr19	27801814	59128983	1775	0.21227284
GBM-4	chr20	0	26225145	2417	0.20378578
GBM-4	chr20	29843182	63025520	2602	0.19883271
GBM-4	chr6	61000000	171115067	7460	-0.3508352
GBM-4	chr7	0	51782692	4238	0.27776182
GBM-4	chr7	51782692	52598931	87	1.73613167
GBM-4	chr7	52598931	54308149	166	0.25258893
GBM-4	chr7	54308149	55929340	113	1.59497511
GBM-4	chr7	55929340	57789531	74	0.2445792
GBM-4	chr7	61760895	159138663	5555	0.27552706
GBM-4	chr9	0	38771460	3182	-0.3398405
GBM-4	chrX	2600587	59336852	2939	0.20870879
GBM-4	chrX	65125757	155270560	3657	0.19024354
GBM-5	chr10	0	38685231	3186	-0.2915107
GBM-5	chr10	42810783	135534747	6466	-0.2906111
GBM-5	chr12	126170124	133851895	745	-0.3216546
GBM-5	chr14	20213937	60091062	2922	-0.3071399
GBM-5	chr14	94957122	99352525	476	-0.2870158
GBM-5	chr19	0	24556461	1585	0.13345
GBM-5	chr19	27801814	59128983	1775	0.18974163
GBM-5	chr4	54390405	55777663	143	1.01741278
GBM-5	chr7	0	54798944	4528	0.27422626
GBM-5	chr7	54798944	55752011	67	1.72673476
GBM-5	chr7	55752011	57789531	84	0.19038256
GBM-5	chr7	61760895	159138663	5554	0.26815701
GBM-5	chr9	17628603	21088482	296	-0.3361606
GBM-5	chr9	21088482	22682383	94	-1.0789731
GBM-5	chr9	22682383	34545936	768	-0.3717474
GBM-5	chrX	147901155	148426388	36	-1.4507785
GBM-6	chr1	144028314	249250621	6800	0.09455409
GBM-6	chr10	0	38685231	3187	-0.2869212
GBM-6	chr10	42810783	135147710	6454	-0.2869258
GBM-6	chr11	0	3258660	263	-0.148476
GBM-6	chr11	20724744	32499712	815	-0.3416675

GBM-6	chr12	0	34768168	3003	0.11108959
GBM-6	chr12	38416139	133851895	6369	0.11253951
GBM-6	chr14	20213937	107349540	6293	-0.3162971
GBM-6	chr15	62635533	102531392	3208	0.10335293
GBM-6	chr19	0	22636339	1464	-0.1792643
GBM-6	chr19	27801814	59128983	1775	-0.2317279
GBM-6	chr22	27716447	51304566	1865	-0.3892314
GBM-6	chr4	53636302	55950410	196	0.96617809
GBM-6	chr5	49646456	68688185	1401	0.09279954
GBM-6	chr5	145413427	180915260	2930	0.36829613
GBM-6	chr7	0	57789531	4679	0.1655743
GBM-6	chr7	61760895	159138663	5555	0.16256829
GBM-6	chr8	8101641	43646413	2934	0.09385553
GBM-6	chr8	47060977	50079299	122	0.10755109
GBM-6	chr9	0	36789658	3039	0.13667238
GBM-6	chr9	0	36789658	3323	0.13667238
GBM-6	chr9	38278780	38771460	50	0.22479547
GBM-6	chr9	71034203	141213431	5249	0.10609303
GBM-7	chr1	144028314	249250621	6799	0.11953293
GBM-7	chr10	0	38685231	3187	-0.1555722
GBM-7	chr10	42736181	135534747	6465	-0.107969
GBM-7	chr11	48030640	51530241	84	0.11935612
GBM-7	chr7	0	54844436	4531	0.13791326
GBM-7	chr7	54844436	55297642	35	1.58419883
GBM-7	chr7	61760895	159138663	5555	0.11611944
GBM-7	chrX	0	59336852	2945	0.13798866
GBM-7	chrX	65125757	95045222	673	0.07154009
GBM-7	chrX	95045222	155270560	2984	0.15359572
GBM-7	chrY	2655180	10077460	185	-2.2786016
GBM-7	chrY	13656660	19567718	388	-2.135462
GBM-7	chrY	20837553	24522333	214	-2.1684209
GBM-8	chr1	0	10305461	776	-0.1986077
GBM-8	chr1	10305461	13989759	249	0.83895469
GBM-8	chr10	0	38685231	3186	-0.2342931
GBM-8	chr10	42810783	135534747	6463	-0.2459807
GBM-8	chr11	0	51530241	4164	0.09724915
GBM-8	chr11	87520390	135006516	3964	-0.1802706
GBM-8	chr13	19263735	37123178	1447	-0.3191019
GBM-8	chr14	20213937	24394659	431	0.09102711
GBM-8	chr14	24394659	107349540	5863	-0.2720035
GBM-8	chr16	0	5369459	325	-0.144346
GBM-8	chr19	23476871	24556461	78	0.23581218
GBM-8	chr19	27801814	59128983	1775	0.15619838

GBM-8	chr20	0	26225145	2416	0.22893269
GBM-8	chr20	29843182	63025520	2602	0.2007062
GBM-8	chr22	16079545	51304566	2737	-0.3624178
GBM-8	chr5	49646456	68688185	1401	0.09443038
GBM-8	chr7	0	54308149	4492	0.26490973
GBM-8	chr7	54308149	55408537	86	1.46692967
GBM-8	chr7	55408537	57789531	101	0.24015945
GBM-8	chr7	61760895	159138663	5555	0.25390834
GBM-8	chr9	0	20344642	2032	-0.2349665
GBM-8	chr9	20344642	26809100	429	-1.0085449
GBM-8	chr9	135347758	141213431	449	-0.1791288
