# Supplementary figures and images for: Farewell to GBM-O: Genomic and transcriptomic profiling of glioblastoma with oligodendroglioma component reveals distinct molecular subgroups
Source: Acta Neuropathol Commun. 2016 Jan 13;4:4. doi: 10.1186/s40478-015-0270-7 (PMC4711079; doi:10.1186/s40478-015-0270-7)

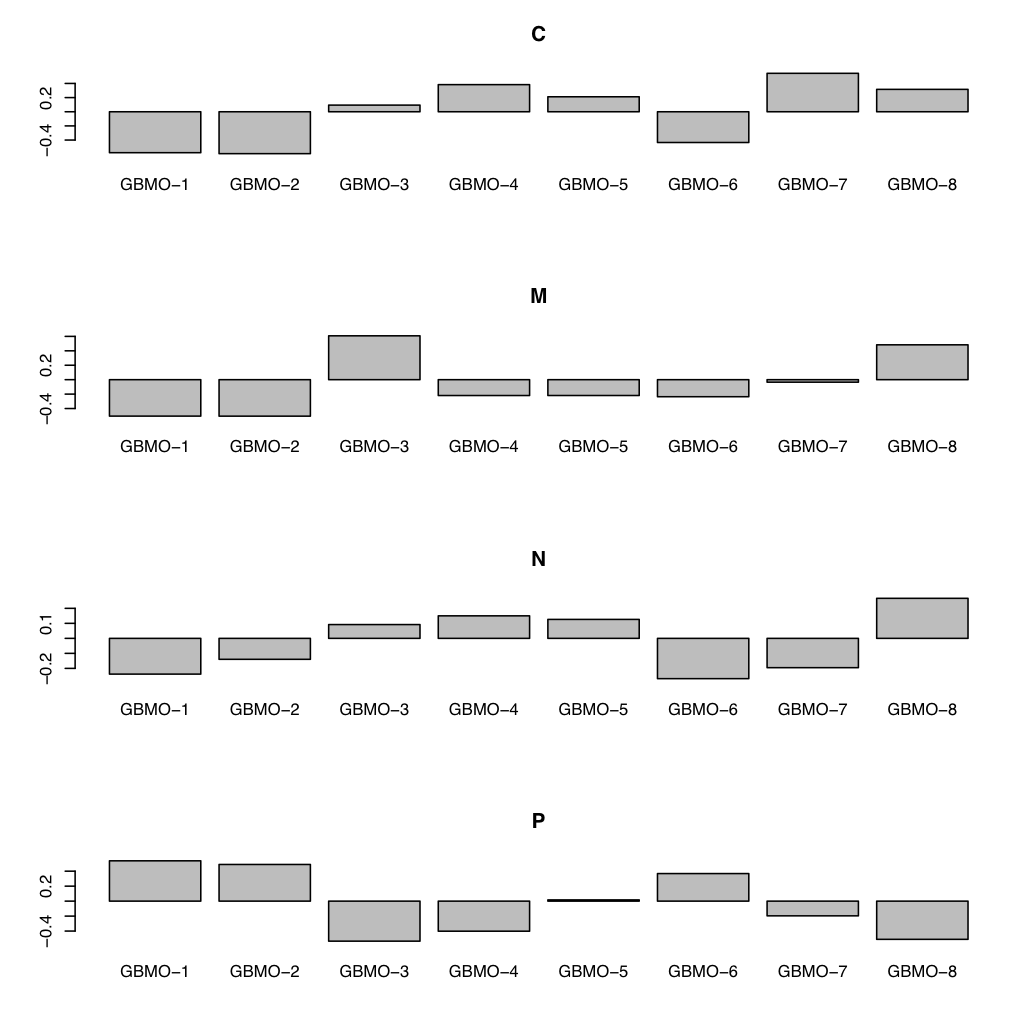

Supplement: Additional file 5: Figure S1. — Expression histogram. (TIFF 3115 kb) [file 40478_2015_270_MOESM5_ESM.tiff]
